# Track and dose-average LET dependence of Gafchromic EBT3 and MD-V3 films exposed to low-energy photons

**DOI:** 10.1038/s41598-020-59233-7

**Published:** 2020-02-11

**Authors:** G. Massillon-JL

**Affiliations:** Instituto de Física, Universidad Nacional Autónoma de México, 04510 Coyoacan Mexico City, Mexico

**Keywords:** Applied physics, Nuclear physics, Techniques and instrumentation

## Abstract

Gafchromic films are widely used in radiotherapy using photons, electrons and protons. Dosimetric characteristics of the films in terms of beam-quality is of great importance for a better evaluation of the absorbed-dose in the clinic. In proton-therapy, film’s response has been reported in terms of track-average, *L*_Δ,*T*_, or dose-average, *L*_Δ,*D*_, linear energy transfer (LET), concluding that *L*_Δ,*D*_ is a more reliable parameter than *L*_Δ,*T*_. Nonetheless, in photon-beams, the film’s response is generally scrutinised in terms of photon-energy. This work aimed at investigating, the total (TEF) and secondary (SE) electron fluence produced in EBT3 and MD-V3 films exposed to 20 kV-160 kV x-ray and ^60^Co beams and their corresponding *L*_Δ,*T*_ and *L*_Δ,*D*_ to determine their influence on the film’s relative-efficiency, *RE*_*Film*_. Regardless the film-model, at energies below 100 keV, *L*_Δ,*D*_ for TEF are about 1.7 to 2.5 times those of *L*_Δ,*T*_ while for SE they are relatively similar (8–29%). For ^60^Co-gamma, *L*_Δ,*D*_ for TEF and SE are approximately 9 and 4 times *L*_Δ,*T*_, respectively, which implies that *L*_Δ,*D*_ is more important for high-photon energies. Independent of the electron-fluence and film-model, *RE*_*Film*_ is almost constant at low average-LET, rapidly increases and thereafter steadily rises with average-LET. The *RE*_*Film*_−*LET* curve indicated that *L*_Δ,*D*_ is more sensitive to small change than *L*_Δ,*T*_ and if it is evaluated for SE, it would even be more appropriate to better describing the dosimeter response induced by photons in terms of ionization-density instead of *L*_Δ,*T*_ for TEF, as generally done. Based on these results, once can conclude that the effect of the average-LET on the film’s response should be considered when use for clinical-dosimetry using photons and not only the energy.

## Introduction

After the introduction of the linear energy transfer (LET) concept by Zirkle and colleagues^1^, the international commission on radiation units and measurement (ICRU)^2^ has adopted two non-stochastic quantities to describe the quality of an ionising radiation beam: the track-average LET, *L*_Δ,*T*_, which describes the average energy lost by charged particles due to collisions per distance travelled with energy transfers less than some specific Δ value and the dose-average LET, *L*_Δ,*D*_, that corresponds to the average LET associated to the absorbed dose distribution^2^. Since then, *L*_Δ,*T*_ has been conventionally used to quantify the radiation-induced effect in any biological^3–6^ and physical^7–12^ systems. During the last few years, the dose-average LET, *L*_Δ,*D*_ has received some particular importance due to the extensive use of protons for radiotherapy treatment where there is an interest for including, into the treatment planning system, parameters that are clinically and biologically relevant^13–15^. In terms of macroscopic dosimetric parameters, Paganetti and colleagues have reported that *L*_Δ,*D*_ is more suitable for studying the biological effectiveness instead of *L*_Δ,*T*_^13,15^ while Guan and collaborators suggested the use of both quantities, but at different energy intervals^14^.

Due to their high spatial resolution, Gafchromic films are widely used for quality assurance and/or absorbed dose distribution measurements in radiotherapy procedures using photons^16–19^, electrons and protons^7,12,20–27^. However, the dosimetric characteristics of the films in terms of radiation beam quality is of great importance for a better evaluation of the absorbed dose in the clinic. In proton-therapy beams, several groups have studied radiochromic film’s response in terms of LET^12,15,23,26,28^. Interestingly, similar to biological systems, Reinhardt and collaborators have stated that *L*_Δ,*T*_ is not suited to describe the LET-dependence of Gafchromic film, suggesting *L*_Δ,*D*_ instead as a more reliable parameter to analyse the film’s response after exposure to protons^12^. Contrary to protons, radiochromic film’s response induced by photons is generally scrutinised in terms of the average or effective photon energy despite of their use to measure dose profile outside of radiation fields in new modern radiotherapy techniques where the absorbed dose is generally low and there exists a large contribution of low photon energy. For example, it has been reported that the relative fraction of low photon energy (<100 keV) increases inversely with photon dose in low-dose regions of three-dimensional conformal radiotherapy (3D-CRT) and intensity-modulated radiation therapy (IMRT) fields^29^. Besides, increase up to 12% on the response of TLD-100 situated outside of the field was observed comparing to 1% variation within the treatment field of a 6 MV x-ray linear accelerator^30^. Furthermore, secondary electrons generated by low photon energies have been found to be mostly of energies below 10 keV and have track-average LET as high as of 19 keV*μ*m^−1^ and 9 keV*μ*m^−1^ in LiF:Mg,Ti and liquid water, respectively similar to those of 76 MeV–120 MeV ^3^He ions^8^.

Recently, we have investigated the film response’s relative efficiency, *RE*, (ratio of absorbed-dose required to produce the same net optical density (netOD) by ^60^Co gamma and by x-rays) of EBT3 and MD-V3 films exposed to low energy x-rays^31^. Following the biological approach and considering the non-linearity of the film’s response with absorbed dose, *RE* was defined as the ratio of the absorbed dose deposited by ^60^Co gamma with respect to that imparted by x-rays that produces the same response^31^. The *RE* results suggested that 4 and 3 times absorbed dose from ^60^Co gamma rays need to be delivered to the EBT3 and MD-V3 sensitive volumes, respectively to produce the same netOD than when exposed to 20 kV x-rays. Besides, *RE* was found to be independent of the absorbed dose imparted during the irradiation^31^. A possible explanation was that the quantity of ionization density (i.e. LET) produced by a given beam quality might probably be the main responsible for the activation of colour centres which gives rise to the polymerization process, regardless the amount of absorbed dose imparted during the irradiation process^31^. To support that statement, the relationship between the film response and LET should be established. Besides, in a recent study about the secondary electron (SE: electrons generated by electron-electron interactions) spectra generated in LiF by low photon energies and their corresponding track-average LET, we found that SE spectra could contribute to a better understanding of the dosimeter response induced by photon beams in terms of ionization-density^8^. Thus, to determine the role of the ionisation density in the polymerization process, investigation on the average-LET dependence of radiochromic film’s response after exposure to photons is needed.

In this work, we investigated the linear energy transfer (LET) dependence of Gafcrhomic EBT3 and MD-V3 films exposed to 20 kV-160 kV x-ray and ^60^Co gamma beams. In particular, we calculated both LET quantities: the track-average LET, *L*_Δ,*T*_, and the dose-average LET, *L*_Δ,*D*_, of the total electron fluence (TEF) and SE spectra generated within the film’s sensitive volumes by the photon beams and determined their influence on the film’s relative efficiency, *RE*_*Film*_, recently reported. The results of this study might have an impact in the clinic due to de dependence of the films on radiation beam quality.

## Methods

### Electron fluences, absorbed dose and Track-average LET calculation within the sensitive volume of the Films

Dosrznrc and Flurznrc modules from the EGSnrc Monte Carlo (MC) code^32^ were used to calculate the absorbed dose as well as the electron fluence spectra, respectively. In the EGSnrc code, it is possible to score the “total electron fluence” (TEF) where all electrons generated by photons and by electron–electron interactions are counted; the “primary electrons” (PE) that compute all electrons generated by photons during the interaction and “secondary electrons” (SE) which include all electrons produced due to electron–electron interactions. We are particularly interested on the SEs since our previous work for LiF suggested that the LET of SEs better describes the experimental observation of the dosimeter response than the LET of TEF^8^. This is explained by the fact that SEs have very short range (order of nanometers) and cannot move far away from their origin, so consequently deposit their energy locally and activate more colour centers within the film sensitive volume. The simulation was performed considering the same parameters selected in our previous work^8^, i.e. 512 keV and 1 keV electron transport cut-off (ECUT) and photon transport cut-off (PCUT), respectively with Bound Compton, spin effects and Rayleigh scattering turned on. Table 1 displays the characteristic of the radiation beams used and the corresponding x-ray spectra is reported elsewhere^33^ (see Fig. 3 in ref. ^31^). The cross-section database generated by the XCOM package^34^ was considered. The geometry was exactly the same as the experimental setup recently reported^35^. This corresponds to 61 cm source to film distance (SFD) and 8.2 cm diameter field size in the case of the x-ray beams and 100 cm SFD and 14 × 14 cm^2^ field size for the ^60^Co. For the ^60^Co gamma simulation, the photon spectrum provided by the EGSnrc code was used instead of the 1250 keV monoenergetic beam. The calculations were made considering the films exposed in air for the x-ray beams and at 4.323 cm depth of a PMMA phantom of 30 cm × 30 cm × 14 cm for the ^60^Co gamma in order to provide charged particle equilibrium. In the simulations, 5 × 10^9^ histories were followed for both the absorbed dose and the electron fluence. Table 2 depicts the chemical composition of the films. The scoring region for the electron fluence and absorbed dose was 1.4 × 1.4 cm^2^. Using the electron fluences, the track-average LET, *L*_Δ,*T*_, and the dose-average LET, *L*_Δ,*D*_, in the film sensitive volume was evaluated using the following relation^36^:

1 $${L}_{\Delta ,T}=\frac{{\int }_{\Delta }^{{E}_{max}}\,{L}_{\Delta }(E)\Phi ({\rm{E}}){\rm{dE}}+S(\Delta )\Phi (\Delta )\Delta }{{\int }_{\Delta }^{{E}_{max}}\Phi ({\rm{E}}){\rm{dE}}+\Phi (\Delta )\Delta },$$

2 $${L}_{\Delta ,D}=\frac{{\int }_{\Delta }^{{E}_{max}}\,{L}_{\Delta }^{2}(E)\Phi ({\rm{E}}){\rm{dE}}+{S}^{2}(\Delta )\Phi (\Delta )\Delta }{{\int }_{\Delta }^{{E}_{max}}\,{L}_{\Delta }(E)\Phi ({\rm{E}}){\rm{dE}}+S(\Delta )\Phi (\Delta )\Delta },$$

where $S(E)$, ${L}_{\varDelta }(E),$ $E\,{\rm{and}}\,\Phi (E)$ are the calculated unrestricted and restricted stopping power^37^, the electron energy and the electron energy fluence, respectively. $S(\varDelta )\Phi (\varDelta )\varDelta$ represents all the electrons that fall below $\varDelta =1\,{\rm{keV}}\,$^36^ due to the lack of accurate electron cross sections at energies below 1 keV^8,38^.

**Table 1:** Beam characteristics used in the simulation.

Beams	Additional filter	1^st^ HVL	2^nd^ HVL	Effective energy
	(mm) Al	(mm) Al	(mm) Al	keV
20 kV	0.2794	0.25	0.39	13.476
50 kV	1.0668	1.13	1.72	23.56
80 kV	2.8702	2.832	4.17	32.3
120 kV	7.112	6.538	8.36	47.93
160 kV	5.2324 + 0.254 Cu	10.4	10.75	67.385
^60^Co				1044.7

**Table 2:** Chemical composition of the films according to the manufacturer.

Element	MD-V3		EBT3	
	Active layer (%): Z_eff_ = 7.63	Overall (%): Z_eff_ = 6.68	Active layer (%): Z_eff_ = 7.46	Overall (%): Z_eff_ = 6.71
H	58.2	38.3	56.5	38.4
Li	0.6	0.0	0.6	0.1
C	27.7	43.9	27.4	43.7
N	0.4	0.00	0.3	0.0
O	11.7	17.7	13.3	17.7
Na	0.5	0.00	0.1	0.0
Al	0.3	0.0	1.6	0.2
S	0.1	0.0	0.1	0.0
Cl	0.6	0.00	0.1	0.0
	0.0	0.00		

### Film response’s relative efficiency, RE_Film_

To quantify the importance of LET on the film response, the weight-averaged relative efficiency, *RE*_*Film*_, recently evaluated for both film models and reported previously^29^ was studied as a function of *L*_Δ,*T*_ and *L*_Δ,*D*_. *RE*_*Film*_ is defined by the following relation^31^:

3 $$R{E}_{film}=\frac{{D}_{Film,{Q}_{0}}(netOD)}{{D}_{Film,Q}(netOD)},$$

where ${D}_{Film,Q}(netOD)$ y ${D}_{Film,{Q}_{0}}(netOD)$ represent the absorbed-dose required to generate the same netOD by x-rays and by ^60^Co gamma, respectively.

## Results

**Figure 1:**
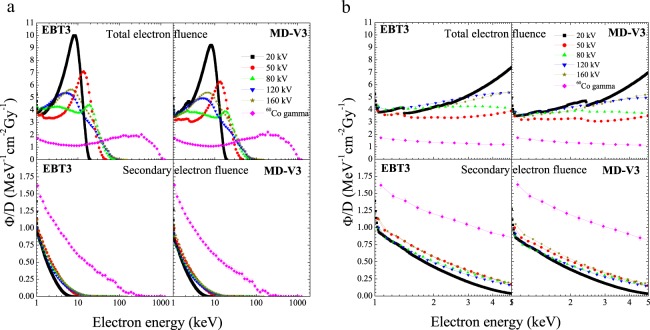
(**a**) Total and secondary electron fluences normalized to the absorbed dose as a function of electron energy for both film models. (**b)** Zoom of (**a**) to better visualize the k-shell electrons.

**Figure 2:**
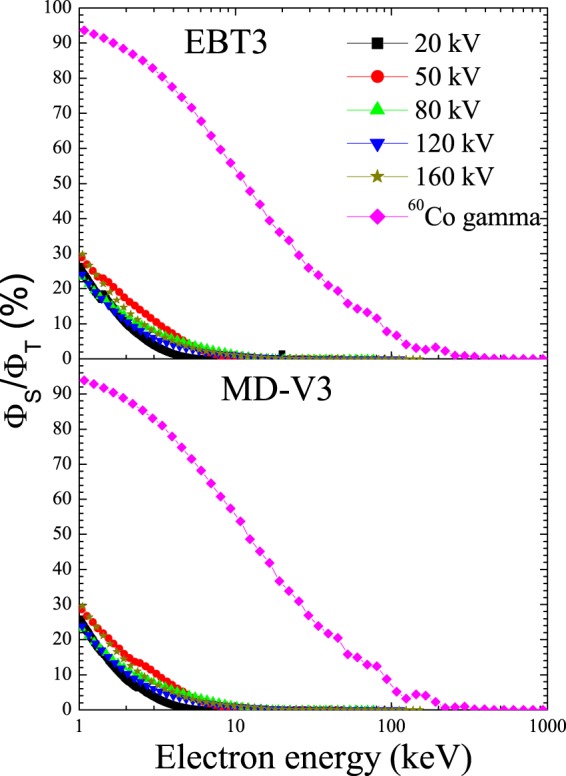
Relative contribution of secondary electron fluences with respect to the total fluence for both film models.

The total fluence (TEF) and secondary electron (SE) spectra normalised to the absorbed dose imparted within the sensitive volume of the films are shown in Fig. 1a for both films. Noted that independent of the film models, except at 80 kV x-rays where two main peaks are depicted, the TEF spectra display only one main peak at all x-rays and the ^60^Co gamma beams. Besides, some small structures are observed at electron energies below 3 keV. These structures correspond to the k-shell electrons generated due to the presence of Na, Al, S and Cl as part of the sensitive volume chemical compositions of both films (see Table 2). Figure 1b depicted a zoom into the low-energy region in order to better visualize the k-shell electron contributions of the elements. Concerning the SE spectra, the fluences steadily decrease as a function of the electron energy and in contrast to the TEF, there is no presence of k-shell electrons. Figure 2 presents the ratio of SE spectra and the TEF. Note that there is a higher contribution of low-energy SE at higher photon energy beams.

**Figure 3:**
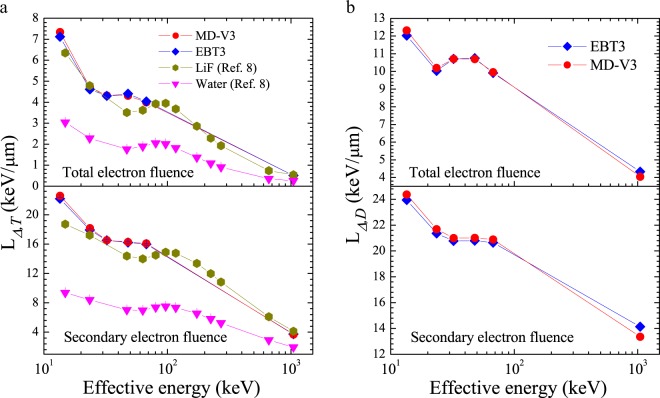
(**a**) Track-average LET generated in both film models as a function of the equivalent photon energy. (**b**) Dose-average LET generated in both film models as a function of effective photon energy.

**Figure 4:**
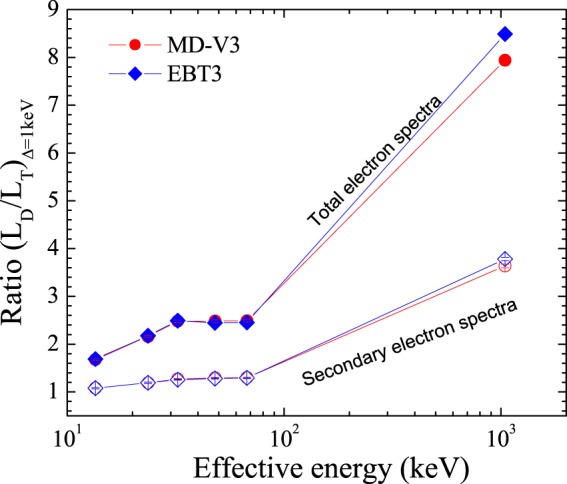
Ratio of dose average relative to track-average LET generated in both film models as a function of effective photon energy.

The track-average LET, *L*_Δ,*T*_, and the dose-average LET, *L*_Δ,*D*_, for the TEF and SE spectra generated in both film models by the photon energy beams and calculated through Eqs. 1 and 2 are displayed in Fig. 3a,b, respectively, whereas their values are reported in Tables 3 and 4. Note that the high LET values of the SE spectra produced by the low photon energies. Figure 4 shows the ratio of *L*_Δ,*D*_. relative to *L*_Δ,*T*_ generated in both film models as a function of effective photon energy. As it can be seen, for a given photon energy beam, the average LET associated to the absorbed dose distribution within both films is much larger than the track-average LET and becomes even more important as the photon energy increases.

**Table 3:** Track-average LET of the Total and SE spectra in EBT3 and MD-V3 situated in air as a function of equivalent photon energy beams. Combined standard uncertainty^39^ of 0.7% (coverage factor *k* = *1*).

*L*_Δ,*T*_ [*keV*/*μm*]				
*Equivalent Energy*	Total electron fluence		Secondary electron fluence	
(keV)	EBT3	MD-V3	EBT3	MD-V3
13.48 ± 0.01	7.12	7.34	22.20	22.60
23.56 ± 0.04	4.61	4.72	17.93	18.18
32.33 ± 0.04	4.30	4.32	16.52	16.54
47.93 ± 0.02	4.40	4.31	16.23	16.28
67.39 ± 0.01	4.04	4.00	15.98	16.11
1044.7	0.51	0.51	3.74	3.67

**Table 4:** Dose-average LET of the Total and SE spectra in EBT3 and MD-V3 situated in air as a function of equivalent photon energy beams. Combined standard uncertainty^39^ of 0.7% (coverage factor *k* = *1*).

*L*_Δ,*D*_ [*keV*/*μm*]				
*Equivalent Energy*	Total electron fluence		Secondary electron fluence	
(keV)	EBT3	MD-V3	EBT3	MD-V3
13.48 ± 0.01	12.03	12.32	23.94	24.36
23.56 ± 0.04	10.03	10.19	21.36	21.69
32.33 ± 0.04	10.71	10.71	20.77	20.99
47.93 ± 0.02	10.75	10.70	20.79	21.02
67.39 ± 0.01	9.91	9.97	20.64	20.88
1044.7	4.33	4.05	14.14	13.35

**Figure 5:**
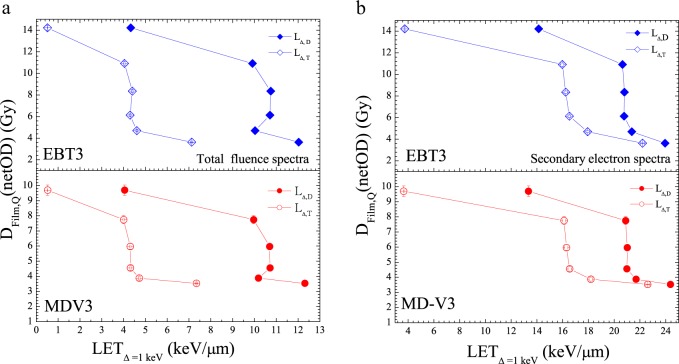
(**a**) Absorbed dose within the film sensitive volume required to generate the same netOD by different beam qualities as a function of LET from the TEF spectra. (**b**) Absorbed dose within the film sensitive volume required to generate the same netOD by different beam qualities as a function of LET from the SE spectra.

**Figure 6:**
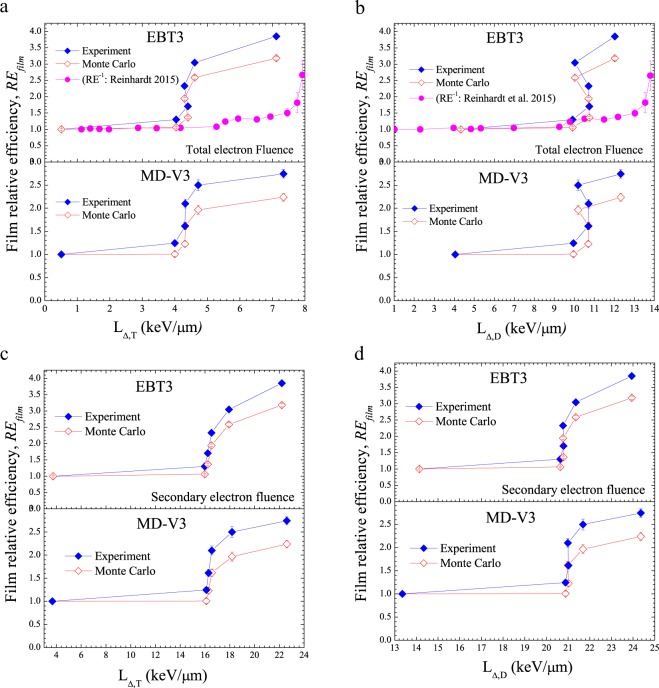
(**a**) Weight-averaged *RE*_*film*_ for both films as a function of track-average LET of the TEF spectra. The EBT3 data of Reinhardt *et al*. refers to protons. (**b**) Weight-averaged *RE*_*film*_ for both films as a function of dose-average LET of the TEF spectra. The EBT3 data of Reinhardt *et al*. refers to protons. (**c**) Weight-averaged *RE*_*film*_ for both films as a function of track-average LET of the SE spectra. (**d**) Weight-averaged *RE*_*film*_ for both films as a function of dose-average LET of the SE spectra.

Figures 5a,b present the absorbed dose within the film sensitive volume required to generate the same netOD, ${D}_{Film,Q}(netOD)$, by different beam qualities as a function of the average-LET from the TEF and SE spectra, respectively. Note that lower is the average-LET, the higher is the absorbed dose needed to produce a certain amount of netOD, regardless the film models. From our previous work^31^, we have shown that independent of the colour channel the relative efficiency, *RE*_*Film*_, is statistically the same for different netOD values. For the red channel for example, the netOD values selected were 0.053, 0.176 and 0.510 for EBT3 and 0.009, 0.023 and 0.095 for MD-V3 (see Tables 3 and 4 as well as Fig. 6a,b from ref. ^31^). Thus, based on this observation a weight-averaged *RE*_*Film*_ was calculated per each photon energy beam. The results for both film as a function of *L*_Δ,*T*_ and *L*_Δ,*D*_, produced by the TEF are shown in Fig. 6a,b, respectively. Whereas, Fig. 6c,d display the result for the SE spectra.

As observed, independent of the film models and electron spectra, *RE*_*Film*_ gradually increases, has an abrupt rising region, thereafter tends to be constant with average LET. Also displayed in Fig. 6a,b is the data published in the literature for EBT3 film exposed to proton beams as a function of *L*_Δ,*T*_ and *L*_Δ,*D*_, respectively. A qualitative agreement can be seen with our results for electrons generated by photons.

## Discussion

### Electron fluences

According to Fig. 1a, the TEF spectra generated in both films display two main peaks at 80 kV x-rays and only one at all others x-ray and ^60^Co beams. The main peak shown at 20 kV and 50 kV x-rays corresponds to the photoelectric process while that at the other energy beams is due to Compton effect. The lower energy peak with broad maxima at 80 kV x-rays is correlated to Compton process while the higher energy one is associated to the photoelectric. Furthermore, four small peaks can be seen at energy below 3 keV in Fig. 1b. These peaks are related to 1.07 keV, 1.56 keV, 2.47 keV and 2.82 keV K-shell electrons of Na, Al, S and Cl, respectively. Note that there is also a more significant peak at energies close to 2.82 keV in the case of the MD-V3 film and close to 1.56 keV for the EBT film. This is presumably associated to the amount of Cl and Al concentrations (see Table 2) presents into the sensitive volume of MD-V3 (0.6% Cl) and EBT3 (1.6% Al) films, respectively. Contrary, the shape of the SE spectra has not revealed any type of photon interaction process with the films, which support our previous statement that the SE do not have memory of their origin^8^. As observed in Fig. 2, the contribution of the SE in the TEF for ^60^Co gamma is around 40–90% at energy between 1 keV and 10 keV regardless the film model, which agrees quite well with the data published recently for LiF:Mg,Ti and liquid water^8^. However, for the x-ray beams, the maximum contribution of SE at 1 keV is less than 30%. This is lower than that observed previously for LiF:Mg,Ti and liquid water exposed to 20 kV narrow x-ray beam. This difference could possibly be associated to the photon energy spectra since in the previous work, the x-ray beams were highly filtrated whereas in this work, the beams have low filtrations. Such observation suggests that the number of SE generated during the interaction process is most likely related to the photon energy spectra rather than the medium. That is, higher photon energy would generate primary electrons (PE) with high energy enough to produce multiple SE cascades along their paths. While PE generated by low photon energy beams would loss all their energy at short distances and consequently few SE cascades will be produced. Comparing the two film models, noted that for a similar area of 1.4 ×1.4 cm^2^, the EBT3 film sensitive volume is almost twice that of the MD-V3 due to the active layer thickness (see Fig. 1 in ref. ^31^). Nevertheless, the electron spectra produced in EBT3 film are greater than those in MD-V3 film by only 4–10% and 2–12% for the TEF and SE spectra, respectively being smaller at higher photon energy beams. This small discrepancy between the two films suggests that for a same sensitive volume, more electrons would be generated within the MD-V3 film caused by its chemical composition.

### The track-average, *L*_Δ,*T*_, and dose-average, *L*_*Δ,D*_, LET within the film sensitive volumes

As observed in Tables 3 and 4 as well as Fig. 3a,b, qualitatively both the track-average, *L*_Δ,*T*_ and the dose-average, *L*_Δ,*D*_ LET, for the TEF spectra generated in both film models steadily decrease with the effective photon energy beams to a minimum at ~32 keV, later rise, reach a maximum at ~48 keV and then decrease toward ^60^Co gamma. Such behaviour has been explained in our previous work and interpreted as a consequence of the competition between Compton and Photoelectric effects^8^. Whereas, for the SE spectra both average-LET quantities displayed a relatively slight plateau between 32 keV and 67 keV instead of a local minimum before decreasing to ^60^Co gamma energies. This feature contrasts with the data published previously for LiF and liquid water^8^ also displayed in Fig. 3a. This could presumably be related to the soft x-ray beams, i.e. less additional filtration, used in this study. For example, as Fig. 3a shown, in our previous study where hard x-ray beams, i.e. high additional filtration, were used^8^, the energy at which *L*_Δ,*T*_ reaches the minimum was ~50 keV effective energy, independent of the medium. Furthermore, the energy at which *L*_Δ,*T*_ is maximum is also shifted toward higher energy for the hard x-ray beams comparing to the soft beams (~100 keV for hard beam vs ~48 keV for soft beam). This suggests that the energy where *L*_Δ,*T*_ reaches a minimum or a maximum is most likely related to the photon energy spectra rather than the medium of interaction. Besides, the *L*_Δ,*T*_ values obtained in this work are greater than those reported for LiF and liquid water exposed to hard x-ray beams. This can be explained by the fact that photon beams with lower energies, transfer less kinetic energy to the PEs that generate low velocity SE which produce large amounts of ionization density.

The *L*_Δ,*T*_ values for the SE spectra are significantly larger than those for the TEF spectra by about 3 to 7 times, depending on the photon energy beam. This result agrees quite well with data reported previously^8^. Whereas the *L*_Δ,*D*_ values for the SE are greater than those for the TEF spectra by only 2 to 3 times. This suggests that *L*_Δ,*D*_, better reflects the high contribution of the SE spectra generated by the photons as seen in Fig. 1a. On the other hand, *L*_Δ,*T*_ of the SE and TEF spectra generated in both film models by the x-ray beams studied in this work are approximately 4 to 6 and 8 to 14 times those produced by ^60^Co gamma ray, respectively. While for the *L*_Δ,*D*_ of the SE and TEF spectra, these differences are about 1.45 to 1.8 and 2 to 3 times those produced by ^60^Co gamma rays, respectively. Consequently, at a given absorbed dose value, higher film response should be expected when exposed to low photon energy than high energy beams. Such statement is supported by data reported recently where about 4 and 3 times the absorbed dose from ^60^Co gamma rays were found to be necessary for producing the same response in EBT3 and MD-V3, respectively than when exposed to 20 kV x-rays^31^.

Comparing the *L*_Δ,*D*_ with *L*_Δ,*T*_, it can be seen in Fig. 4 that for the SE spectra, they are relatively similar (differences of ~8% to ~29%) at effective energies below 100 keV, independent of the film models. This observation is particularly interesting since it supports our hypothesis regarding the role of SE generated by photons on the ionization process during the radiation interaction with matter. This implies that the calculation of *L*_Δ,*T*_ for SE generated by low photon energies is, to some extent, analogous to assessing the average LET associated to the absorbed dose distribution deposited within the medium due to these spectra. Whereas at ^60^Co gamma, *L*_Δ,*D*_ is approximately 4 times the *L*_Δ,*T*_ value. Regarding the TEF, the ratio slowly increases at energies below 100 keV from ~1.7 to 2.5 and rapidly increases to a value of 9 for ^60^Co gamma. This suggest that for both spectra (TEF and SE), the *L*_Δ,*D*_ is much more important at high photon energy beams. This observation is in agreement with data for liquid water from Table 1 reported by ICRU^2^ where values for this ratio of 2.08, 5.53, 31.58 and 31.36 were obtained due to 50 kV, 200 kV, 22 MV x-rays and ^60^Co gamma, respectively at $\Delta =100\,eV$. Such an agreement proposes that the relation between *L*_Δ,*D*_ and *L*_Δ,*T*_ is most likely correlated to the radiation beam quality rather than the medium.

### Film response’s relative efficiency, RE_Film_, versus track and dose average LET

Noted that the absorbed dose within the film sensitive volume required to generate the same netOD, ${D}_{Film,Q}(netOD)\,$^31^, displayed in Fig. 5a,b steadily decreases at low average-LET, rapidly falls and thereafter tends to be constant as *L*_Δ,*D*_ or *L*_Δ,*T*_ increases, independent of the film models. The almost no variation of the ${D}_{Film,Q}(netOD)$, at certain *L*_Δ,*D*_ or *L*_Δ,*T*_ values could presumably interpreted as a saturation effect caused by the high-ionization density generated by low-energy secondary electrons. Consequently, this could possibly help for explaining the under-response observed for radiochromic film at low photon energy beams. Such observation coincides with the hypothesis that the activation of colour centres that gives rise to the polymerization process within the film sensitive volume is strongly governed by the amount of ionisation density produced by a given beam quality.

With respect to the *RE*_*Film*_ shown in Fig. 6a,d, it can be seen that regardless the film models and the electron fluence spectra, *RE*_*Film*_ is almost constant at low average-LET values, has a rapid increase and after rises slowly as the average-LET increases. This feature is due to the results depicted in Fig. 5a,b and also suggests that in low photon energy region, small variation on the average-LET will produce big change in the film’s response. Besides, in contrast to the data displayed Fig. 6a,c, a small difference between both films model can be observed in the rapid rising region of Fig. 6b,d when the *L*_Δ,*D*_ is considered. This difference is even more remarkable in Fig. 6d for the SE spectra. This means that the *L*_Δ,*D*_ is more sensitive to small change than *L*_Δ,*T*_ and if it is evaluated for SE spectra, it can even be more appropriate to better describing the dosimeter response induced by photon beams in terms of ionization-density instead of *L*_Δ,*T*_ for TEF, as generally done. Also shown in Fig. 6a,d are the *RE*_*Film*_ results obtained through Monte Carlo simulation as a function of average-LET. Note that independent of the film models and electron fluence, the larger is the average-LET, the larger is the difference between the experiment and the MC simulation which was expected according to the results reported in ref. ^31^.

Comparing our result with data reported in the literature for EBT3 film exposed to proton beams^12^, it can be seen in Fig. 6a,b that independent of the radiation beam quality (electrons generated by photons or protons), the shape of the *RE*_*Film*_*-*LET curve is quite similar. Figure 6a indicates that *RE*_*Film*_ varies slowly (variation of ~30%) at *L*_Δ,*T*_ below 4 keV/μm and 7 keV/μm for the TEF spectra and protons, respectively before rising abruptly. While Fig. 6b shows a relatively flat region at *L*_Δ,*D*_ values below 11 keV/μm and 13 keV/μm for the TEF spectra and protons, respectively. Noted that independent of the average-LET, the onset of the rapid increasing region of *RE*_*Film*_ occurs at smaller value for electron than for protons. Such a feature could probably due to the differences in the spatial distribution of the electron spectra generated within the film sensitive volume by both radiation beam qualities at the microscopic level. This suggests that the high ionization density produced by the proton close to its trajectory through the generation of low-energy secondary electron cascades might contribute to larger film’s response than when exposed to photons. Interestingly, this observation is consistent with thermoluminescent relative efficiency, *RE* (equivalent to the inverse of *RE*_*Film*_), results reported previously for low-temperature glow-peaks of LiF:Mg,Ti exposed to several intermediate energy ion beams (see Fig. 2a,b in ref. ^9^) where light particles were observed to have smaller *RE* values than heavier one for a given average-LET value. Contrary to data in Fig. 6a, where differences in *L*_Δ*,T*_ are observed between electrons and protons at low LET, according to Fig. 6b, for *L*_Δ*,D*_ values below 10 keV/μm, *RE*_*Film*_ appears to be independent of the particles, which suggest that the absorbed dose deposited by the proton along the track is mainly due to secondary electrons. This could be understood by the fact that for the track-average LET, each secondary electron is weighted statistically equal while for the dose-average LET, the weight of each track length is based on its contribution to the absorbed dose along the proton path^40^.

## Conclusion

This work has investigated the total fluence (TEF) and secondary electron (SE) spectra generated in Gafcrhomic EBT3 and MD-V3 films exposed 20 kV-160 kV x-ray and ^60^Co gamma beams as well as their corresponding track-average, *L*_Δ,*T*_, and dose-average, *L*_Δ,*D*_, LET in order to determine their influence on the film’s relative efficiency after exposure to low photon energies. In particular, the film response’s relative efficiency, *RE*_*Film*_, has been evaluated as a function of *L*_Δ,*D*_ and *L*_Δ,*T*_. The results support the hypothesis that the activation of colour centres that gives rise to the polymerization process within the radiochromic film sensitive volume is strongly governed by the amount of ionisation density produced by a given beam quality. We also showed that the *L*_Δ,*D*_ is more sensitive to small change than *L*_Δ,*T*_ and if it is evaluated for SE spectra, it would even be more appropriate to better describing the dosimeter response induced by photon beams in terms of ionization-density instead of *L*_Δ,*T*_ for TEF, as generally done. Based on the results of this work, once can conclude that the impact of the average-LET on the Gafchromic film’s response should be taken into account when use for clinical dosimetry using photons.
